# Functional and evolutionary analysis of Korean bob-tailed native dog using whole-genome sequencing data

**DOI:** 10.1038/s41598-017-17817-w

**Published:** 2017-12-11

**Authors:** Daehwan Lee, Dajeong Lim, Daehong Kwon, Juyeon Kim, Jongin Lee, Mikang Sim, Bong-Hwan Choi, Seog-Gyu Choi, Jaebum Kim

**Affiliations:** 1 Department of Biomedical Science and Engineering, Konkuk University, Seoul, 05029 South Korea; 2 National Institute of Animal Science, Wanju, 55365 South Korea; 3 Institute of Conservation Gyeongju Donggyeong Dog, Dongguk University, Gyeongju, 38170 South Korea

## Abstract

Rapid and cost effective production of large-scale genome data through next-generation sequencing has enabled population-level studies of various organisms to identify their genotypic differences and phenotypic consequences. This is also used to study indigenous animals with historical and economical values, although they are less studied than model organisms. The objective of this study was to perform functional and evolutionary analysis of Korean bob-tailed native dog Donggyeong with distinct tail and agility phenotype using whole-genome sequencing data by using population and comparative genomics approaches. Based on the uniqueness of non-synonymous single nucleotide polymorphisms obtained from next-generation sequencing data, Donggyeong dog-specific genes/proteins and their functions were identified by comparison with 12 other dog breeds and six other related species. These proteins were further divided into subpopulation-specific ones with different tail length and protein interaction-level signatures were investigated. Finally, the trajectory of shaping protein interactions of subpopulation-specific proteins during evolution was uncovered. This study expands our knowledge of Korean native dogs. Our results also provide a good example of using whole-genome sequencing data for population-level analysis in closely related species.

## Introduction

Rapid advances in next-generation sequencing technologies has enabled the analysis of animal populations at the DNA sequence level^1^. This trend has led to the launch of many large-scale population-based genome projects such as the 1000 bull genomes project^2^, Drosophila population genomics project^3^, 1000 genomes project^4^, and UK10K genome project^5^. In addition, many smaller-scale population studies have been conducted to discover unique genomic characteristics of a specific population within a species. For example, divergence time between polar bear and brown bear has been identified through sequencing of 89 polar bear and brown bear individuals^6^. Different phenotypic characteristics of goat in coat color, body size, cashmere traits, and high altitude adaptation have been identified by using eight goat individuals^7^. Sequencing data of 133 individuals of rhesus macaque have been obtained to understand evolutionary and behavioral features in relation to human health and disease^8^. Milk production and curly coat trait-associated variants have been identified through sequencing of 234 bulls from the 1000 bull genomes project^9^.

Dogs are important domestic animals with a long history of preservation and breeding for preferable characteristics such as body size, shape, and color. Therefore, many studies have been performed on dog populations^10^. For example, Thai population of Pomeranian dogs have been studied at population-level to investigate patellar luxation^11^. Traits related to adaptation to high-altitude hypoxia have also been studied in six dog breeds based on whole-genome sequencing^12^. Nine Chow Chows from China with 37 published canid whole-genome sequencing data were used to characterize the origin of East Asian dog breeds^13^. In Korea, there are many different native dog breeds, including Donggyeong dog, Jindo dog, Sapsali, and Pungsan dog. Among them, Donggyeong dog is a Korean bob-tailed native dog without a tail or with relatively short tail than other dogs. It is important because of its unusual shape of a tail and superior agility^14^. Donggyeong dog is indigenous to Gyeongju province, Korea. It has the oldest history (recorded in 5~6th century) among native dogs in Korea^15^. To identify genetic diversity, Donggyeong dog breed has been compared to other Korean native dogs such as Jindo dog and Bulgae using microsatellite markers^14^. To preserve unique features of Donggyeong dog, studies have been performed by cloning^15^. Recently, whole-genome sequencing data of 22 Donggyeong dogs was generated, and their unique genetic structure and the development of short tail phenotype were investigated^16^.

The objective of this study was to perform further functional and evolutionary analysis of Donggyeong dog using whole-genome sequencing data^16^. Specifically, genomic features of three different Donggyeong dog populations based on tail length (long, short, and non-tail) were determined and the evolution of protein interactions compared to related dog breeds (a total of 12 breeds) and related species (a total of 10 species) was analyzed. Using sequencing data generated from multiple individuals of selected dog breeds and related species, single nucleotide polymorphisms (SNPs) were identified and Donggyeong dog-specific genes were obtained based on the existence of unique non-synonymous SNPs only present in the population of Donggyeong dog. Functional and variant analyses were then performed for Donggyeong dog-specific genes. These Donggyeong dog-specific genes were further classified by considering the uniqueness of non-synonymous SNPs separately in different Donggyeong dog populations with different tail lengths. Potential functions of these classified gene sets were compared. The mode and extent of changes in protein interactions during evolution were also investigated. This study will expand our knowledge of Korean native dogs. Our results also provide a good example of using whole-genome sequencing data for population-level analysis in closely related species.

## Results

### SNP calling and annotation

A total of 8,667,063, 8,562,023, and 8,902,794 SNPs were obtained from sequence data of Donggyeong dog populations with long-tail (LT), short-tail (ST), and non-tail (NT), respectively (Materials and Methods; Supplementary Table 1). Among them, 1,875,511 (22%), 1,879,258 (22%), and 1,892,844 (21%) SNPs of the LT, ST, and NT population, respectively, were found in the dbSNP database (version 146). Qualities of these SNPs were measured in terms of transition-to-transversion ratio (Ti/Tv). They were 2.03, 2.05, and 2.04 for LT, ST, and NT populations, respectively, similar to those reported in previous studies^17^. These SNPs were classified into synonymous, non-synonymous, splice site, intron, untranslated regions, and intergenic SNPs (Table 1). Most of these SNPs were intergenic variants (71% in LT, ST and NT) or intron variants (27% in LT, 28% in ST and NT). Only a small fraction of these SNPs was found in exons (0.74% in LT, 0.74% in ST, and 0.76% in NT) (Table 1). Results of statistics for SNPs for other species and dog breeds are summarized in Supplementary Table 1.

**Table 1:** Classification of Donggyeong dog SNPs.

	Category	Long tail	Short tail	Non-tail
Exon	Synonymous	34,765 (0.40%)	34,628 (0.40%)	35,713 (0.40%)
	Non-synonymous	29,781 (0.34%)	29,061 (0.34%)	30,625 (0.34%)
Non-Exon	Splice site	6,938 (0.08%)	6,909 (0.08%)	7,174 (0.08%)
	Intron	2,377,868 (27.44%)	2,367,464 (27.65%)	2,449,979 (27.52%)
	UTR	57,514 (0.66%)	57,558 (0.67%)	59,429 (0.67%)
	Intergenic	6,166,978 (71.15%)	6,073,164 (70.93%)	6,326,879 (71.07%)

The fraction to the total number of SNPs in Supplementary Table 1 is shown in parentheses.

### Donggyeong dog-specific genes

A total of 1,814 Donggyeong dog-specific genes were identified considering the existence of non-synonymous SNPs compared to other dog breeds and related species (Materials and Methods, Supplementary Table 2). To find potential functions of these Donggyeong dog-specific genes, Gene Ontology enrichment analysis was performed (Materials and Methods). Among various biological process functions, sensory perception (GO:0007600), sensory perception of chemical stimulus (GO:0007606), sensory perception of smell (GO:0007608), system process (GO:0003008), cellular protein modification process (GO:0006464), neurological system process (GO:0050877), and metabolic process (GO:0008152) were found to be over-represented (Supplementary Fig. 1). The molecular functions, transition elongation factor activity (GO:0003746), translation regulator activity (GO:0045182), receptor activity (GO:0004872), transferase activity (GO:0016740), and catalytic activity (GO:0003824) were identified (Supplementary Table 3). Enriched functions in other categories (molecular function and cellular component) are shown in Supplementary Table 3.

**Figure 1:**
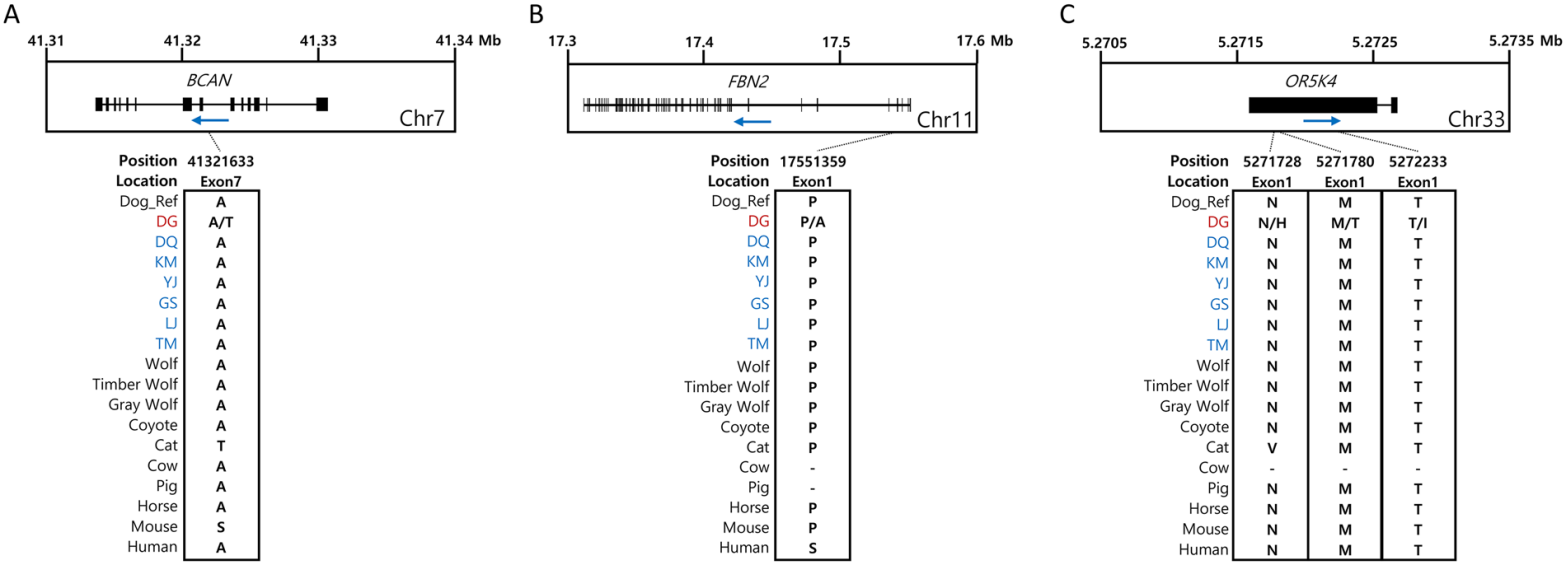
Examples of Donggyeong dog-specific non-synonymous SNPs and consequential amino acid variants. Top panel shows gene structure with the direction of transcription (blue arrow). Bottom panel indicates positions of non-synonymous SNPs and comparison of amino acids among different dog breeds and related species. Two different amino acids corresponding to two nucleotide variants in Donggyeong dog are shown together with a slash delimiter. The dash symbol represents a gap in multiple sequene alignment. DG: Donggyeong dog, DQ: Diquing village dog, KM: Kunming dog, YJ: Yingjiang village dog, GS: German shepherd, LJ: Lijiang village dog, TM: Tibetan mastiff. Other dog breeds not shown here contained the same variant as dog breeds with blue color shown in this figure.

These Donggyeong dog-specific genes were further examined to compare compositions of amino acids to other dog breeds and related species. Among a total of 1,814 genes, three example genes (BCAN, FBN2, and OR5K4) are shown in Fig. 1. In the case of BCAN, the Donggyeong dog population had threonine as an alternative allele with alanine as a reference allele caused by C > T non-synonymous SNP, while other dog breeds and most of other related species had only alanine in the seventh exon (Fig. 1A). FBN2 gene has a non-synonymous SNP in the first exon. This C > G non-synonymous SNP leads to alanine as an alternative allele with proline as reference allele in the Donggyeong dog (Fig. 1B). In OR5K4 gene, only the Donggyeong dog breed had an alternative allele at three positions in the first exon. Other dog breeds and related species had the same reference allele (Fig. 1C).

### Donggyeong dog tail length-specific genes

Donggyeong dog-specific genes were further classified into three gene sets based on the uniqueness of non-synonymous SNPs in these three Donggyeong dog subpopulations (Materials and Methods). A total of 39 genes contained unique non-synonymous SNPs only in the subpopulation with tails (long tail or short tail) while 20 genes contained unique non-synonymous SNPs only in the non-tail subpopulation. If a gene contained non-synonymous SNPs both in the tail and non-tail subpopulations, it was classified as a common gene (a total of 98 genes, Supplementary Table 4). To identify related functions for these gene sets, Gene Ontology enrichment analysis was conducted. In terms of biological processes, 6, 1 and 10 enriched functions were found in tail-specific, non-tail-specific, and common gene sets, respectively. Among them, regulation of nucleobase-containing compound metabolic process (GO:0019219), organelle organization (GO:0006996), and nitrogen compound metabolic process (GO:0006807) were observed only in the tail-specific gene set while translation (GO:0006412) was uniquely observed in the non-tail-specific gene set. In common gene set, several sensory-related functions were found as enriched functions. Details of enriched functions in biological processes, molecular functions, and cellular processes are shown in Supplementary Table 5.

### Protein interactions of Donggyeong dog tail length-specific genes

**Figure 2:**
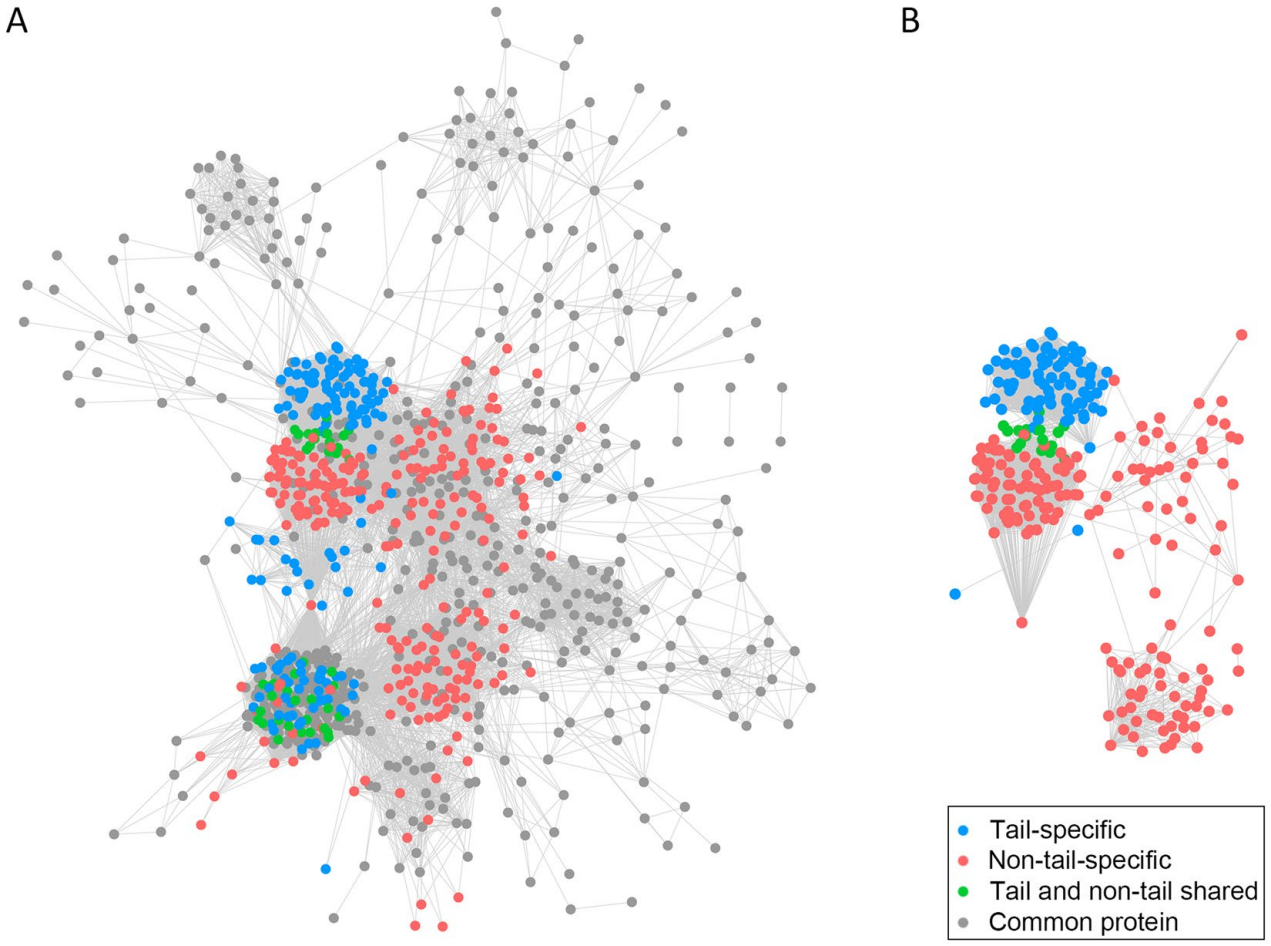
Protein-protein interactions of Donggyeong dog tail length-specific extended protein sets. (**A**) Entire protein-protein interaction network of Donggyeong dog tail length-specific protein sets. (**B**) Tail-specific (blue) and non-tail-specific (red) subnetworks with interactions absent in other related species (cat, horse, pig, and cow). Node colors correspond to the colors used in Supplementary Fig. 2 (blue: the tail-specific proteins not shared with the non-tail-specific and the common proteins, red: the non-tail-specific proteins not shared with the tail-specific and the common proteins, green: shared proteins by the tail-specific and the non-tail-specific proteins excluding the common proteins, and gray: the common proteins). The definition of the protein sets is found in Materials and Methods.

Donggyeong dog tail length-specific gene sets were used to find corresponding protein sets. They were then extended by including additional proteins having direct or indirect interactions with original protein sets (Materials and Methods). Supplementary Fig. 2 shows the Venn diagram of the top 5% proteins of the three extended protein sets based on interaction score (690, 669, and 798 proteins of tail-specific, non-tail-specific, and common protein sets, respectively, Materials and Methods, Supplementary Table 6). A total of 155 proteins were shared by these three protein sets while 35% and 36% of common proteins were also observed in tail-specific and non-tail-specific protein set, respectively. The tail-specific and non-tail-specific protein sets contained 209 common proteins. There was no overlap among seed protein sets used for extension. By using the top 5% of these extended protein sets, protein-protein interaction networks were constructed based on interaction data in STRING database and orthologous protein information in orthoDB (Fig. 2, Materials and Methods). In the constructed network (Fig. 2A, Supplementary Table 7), significantly more numbers of interactions were observed within each of tail-specific and non-tail-specific protein set (Fisher’s exact test, p-value < 2.2e–16). Subnetworks for tail-specific and non-tail-specific protein sets by only considering interactions absent in other related species (cat, horse, pig, and cow) are shown in Fig. 2B. A total of 84 tail-specific proteins and 186 non-tail-specific proteins had 2,407 and 2,331 interactions, respectively. Clustering pattern of interactions with the same type of protein set was more obvious (Supplementary Table 8).

Proteins in tail-specific (blue) and non-tail-specific (red) subnetwork shown in Fig. 2B were further investigated to identify related functions. In the tail-specific subnetwork, several proteins are chemotactic factors and receptors. For examples, IL8 is one of chemotactic factors involved in response to inflammatory stimulus while ACKR3 is an atypical chemokine receptor related to chemokine levels. Among proteins in the non-tail-specific subnetwork, TAC1 encodes four products of the tachykinin peptide hormone family which are thought to function as neurotransmitters known to induce behavioral response. PMCH also produces melanin-concentrating hormone (MCH) that may act as a neurotransmitter or neuromodulater directed toward the regulation of goal-directed-behavior. GH1 is a member of the somatotropin/prolactin family of hormones which play an important role in growth control. In addition, NTS that makes neurotensin with important role in contraction of smooth muscle appeared in the non-tail-specific subnetwork. Other known functions of these proteins are summarized in Supplementary Table 8.

### Evolutionary analysis of protein interactions of Donggyeong dog tail length-specific genes

**Figure 3:**
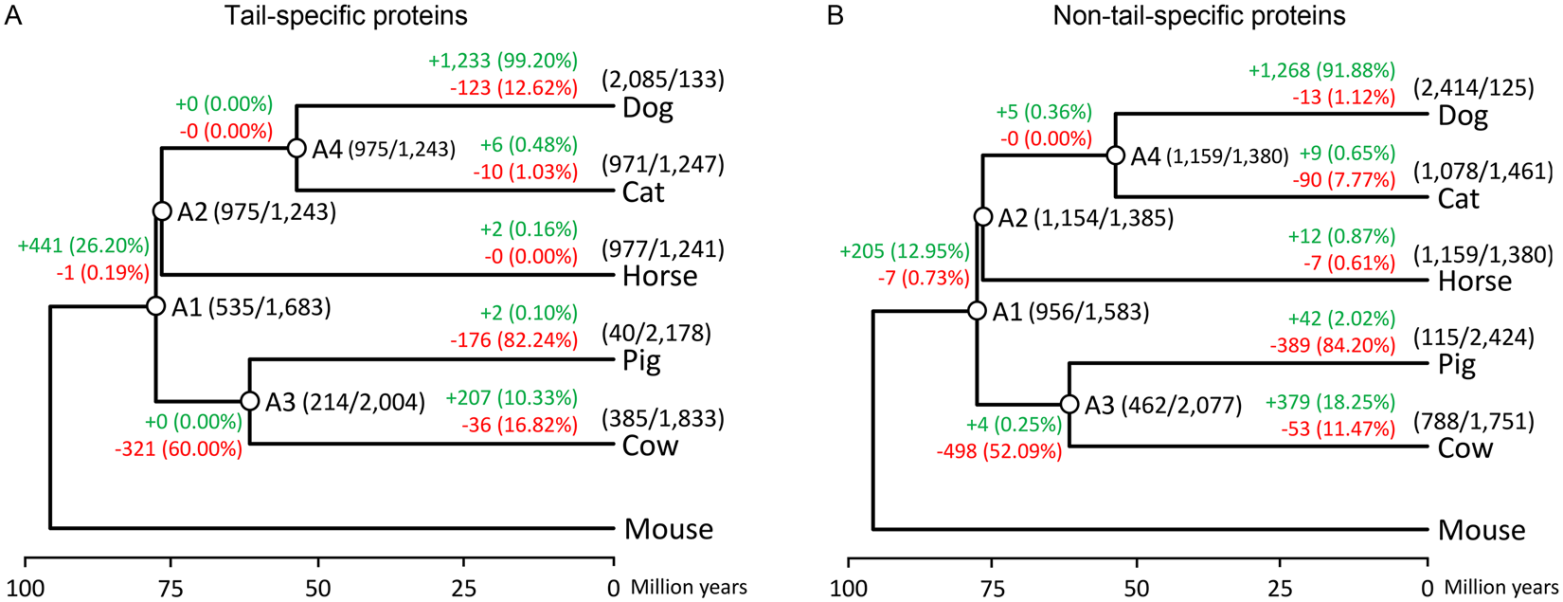
Evolutionary changes in protein interactions among Donggyeong dog tail-specific proteins against a total of 2,218 protein pairs (**A**) and non-tail-specific proteins against a total of 2,539 protein pairs (**B**). Numbers on branches represent the number of newly appeared (in the case of +) or disappeared (in the case of −) protein interactions. Percentages in parentheses indicate fraction against the number of protein pairs without interaction (in the case of +) or the number of protein pairs with interaction (in the case of −). Two numbers next to names of ancestors or above names of descendant species mean the number of protein pairs with or without interaction, respectively. Divergence time among species was obtained from the TimeTree website (http://www.timetree.org/).

The mode and extent of changes in protein interactions during evolution were investigated by considering four related species (cat, horse, pig, and cow) with mouse as an outgroup species. From 2,218 protein pairs of tail-specific extended protein set, interactions in other species were obtained and used to identify the existence of protein interactions in ancestors by using the maximum parsimony algorithm (Materials and Methods, Fig. 3A). From A1 to A2, 26.20% of protein pairs without interaction in A1 had interactions. In addition, significantly larger number of protein interactions (99.20%) were found during evolution from A2 to dog compared to other descendant species of A2. In contrast, many protein interactions disappeared during evolution from A1 to pig and cow. A similar pattern was also obtained from the analysis of non-tail-specific extended protein set (Fig. 3B). The statistical significance of the fraction of interaction changes was investigated based on a randomization test. p-values close to zero were obtained for all branches (Materials and Methods).

## Discussion

In this study, 22 individuals of Donggyeong dog with different tail lengths were investigated to identify the following: (i) breed-specific genes and proteins associated with their unique traits, (ii) their functions, and (iii) evolutionary changes in their interactions by using population and comparative genomics approaches based on whole-genome sequencing data. Donggyeong dogs are very important Korean native dog breeds because of their unusual shape of tail and superior agility^14^. This study was made possible due to the availability of whole-genome sequencing data of mammalian species and other 12 dog breeds. They have been successfully used in other studies investigating evidence of selection during domestication^18^ and reconstruction of the evolutionary history of dogs^19^.

A total of 1,814 Donggyeong dog-specific genes were identified as having non-synonymous SNPs not found in other dog breeds and related species. Majority of them including FBN2 and BCAN were involved in sensory-related functions such as sensory perception (GO:0007600), sensory perception of chemical stimulus (GO:0007606), and neurological system process (GO:0050877) which might affect the agility and appearance of Donggyeong dogs^14^. Among these 1,814 genes, the T gene known to contain a single amino acid variant responsible for short tail length^20^ was not present. This was because Yingjiang village dog included as one of the other 12 dog breeds had the same non-synonymous SNP at the same position in the T gene compared to Donggyeong dog, indicating that some Yingjiang dogs might have short tails.

In our study, the key information for identifying Donggyeong dog-specific genes was the presence of non-synonymous SNPs only in Donggyeong dogs. The non-synonymous SNPs are known to change the amino acid sequence of protein. However, it is very important to collect and use only non-synonymous SNPs that have non-neutral functional effect on phenotype to draw unbiased conclusions. SnpEff ^21^, which is a variant annotation tool, generates additional information, called an impact (or deleteriousness) category, having one of three values, high, moderate, and low. The impact is determined based on the amount of disruption of a gene structure, and high or moderate values indicate the potential effect of a variant on the loss of function or the change of protein effectiveness. In our study, we used only non-synonymous SNPs with the high or moderate impact values as in many recent studies^22–26^. There is also a similar measure, called GERP, to check the phenotypic impact of variants^27–29^. GERP is based on the sequence level conversation of a position with a variant obtained from sequence alignment of multiple species, and assigns higher phenotypic impact on variants observed at more conserved positions.

It is known that biological functions are performed through a cascade of interactions among multiple proteins, not just by a single protein. This fact was reflected in the analysis of protein interactions. Starting from Donggyeong dog tail length-specific proteins identified based on the uniqueness of non-synonymous SNPs, additional proteins directly or indirectly interacting with seed proteins were extracted from the STRING database using a special algorithm called Random Walk with Restart^30^. These extended protein sets including both seed and additional proteins were used to find their interaction patterns and evolutionary characteristics in different Donggyeong dog subpopulations (Fig. 2 and Supplementary Fig. 2).

In the analysis of protein interactions, patterns of interactions of two disjoint protein sets obtained from tail-specific and non-tail-specific populations of Donggyeong dog were examined. As expected, significantly more interactions were observed within tail-specific and non-tail-specific protein sets compared to those observed across different protein sets (Fig. 2A). This pattern became more obvious when protein interactions existing only in dogs were considered (Fig. 2B). This indicates that a separate cluster of proteins interacting with each other might be responsible for different phenotypes of the tail and non-tail subpopulations.

Evolutionary changes of interactions among Donggyeong dog tail length-specific proteins were inspected based on inferred protein interactions in ancestors by the maximum parsimony algorithm (Fig. 3). More protein pairs were found to interact during evolution from the common ancestor of dog, cat, horse, pig, and cow to dog than to other species. This indicates that increased interactions among tail length-specific proteins might have shaped unique traits of these two different Donggyeong dog subpopulations. However, due to limited amount of data in the orthologous protein and protein interaction databases, only 2,218 and 2,539 protein pairs for tail-specific and non-tail-specific protein sets, respectively, were included in this study.

In summary, whole-genome sequencing data of multiple individuals of Donggyeong dogs, other dog breeds, and related species was used to identify non-synonymous SNPs uniquely observed in Donggyeong dogs, and to discover genes and their functions harboring those non-synonymous SNPs. In addition, the mode and extent of evolutionary changes of protein interactions were predicted using population genomic as well as comparative genomic approaches. As the accumulation of whole-genome sequencing data of multiple individuals of various species is accelerated, it is important to comprehensively utilize those data in the perspective of both population genomics and comparative genomics in order to deepen our understanding of species diversity. Our study will contribute to extend the breadth of those studies using whole-genome sequencing data.

## Materials and Methods

### Ethics statement

DNA extraction protocol was approved by the Committee on Ethics of Animal Experiments, National Institute of Animal Science, Republic of Korea (Permit Number: NIAS2015-774). Genomic DNAs were extracted from blood samples obtained from Seoul Grand Park in Republic of Korea with permission. All experiments were performed in accordance with relevant guidelines and regulations.

### Genome sequencing data

The genome sequencing data of Donggyeong dogs with different tail lengths [long tail, N = 7 (~20 coccygeal bones); short tail, N = 5 (5~7 coccygeal bones); non-tail, N = 10 (2~3 coccygeal bones)] was obtained from a recent study^16^. The genome sequencing data of additional 12 dog breeds [Diqing village dog, German shepherd, Kunming dog, Lijiang village dog, Tibetan mastiff, and Yingjiang village dog (N = 10 for each breed), Indian village dog (N = 3), Lebanon village dog (N = 1), modern European breed (N = 1), Nambia village dog (N = 1), Portugal village dog (N = 2), and Vietnam village dog (N = 2)], which was used in another recent study^19^, was downloaded from the NCBI SRA database (http://ncbi.nlm.nih.gov/sra; SRA accession numbers are in Supplementary Table 9). In addition, genome sequencing data of two kinds of wolves (wolf, N = 2; timber wolf, N = 2), and one coyote (N = 1) was generated as follows. Indexed shotgun paired-end libraries with average insert size of 500 bp were generated using TruSeq Nano DNA Library Prep Kit (Illumina, USA) following the standard Illumina sample-preparation protocol. Briefly, 200 ng of gDNAs were fragmented with Covaris M220 (USA) to obtain median fragment size of ~ 500 bp. These fragmented DNAs were end-repaired followed by A-tailing and ligation to indexed adapter (~125 bp adapter). Gel-based selection was performed to select sizes of 550 to 650 bp. PCR amplification was performed in eight cycles. Size-selected libraries were then analyzed with Agilent 2100 Bioanalyzer (Agilent Technologies) to determine size distribution and adapter contamination status. The resulting libraries without adaptor contamination were sequenced on Illumina HiSeq. 2500 sequencing platforms for 2 × 125 bp paired-end sequencing.

### Sequence mapping and SNP calling

The collected genome sequencing data was aligned to Canine reference genome assembly (CanFam3.1) using bowtie2 v2.2.9 with default parameters^12,16,31^. Mapped reads data were converted, sorted, and indexed using SAMtools v1.3.1^32^. Elimination of duplicate reads and generation of quality matrices for mapping were performed using Picard tools v2.1.0 (http://broadinstitute.github.io/picard). Local recalibration and realignment were processed using Genome Analysis Toolkit (GATK; v3.3) framework^33^. A multi-sample SNP-calling procedure was performed to discover SNPs using GATK package. Finally, a filtering step was applied based on GATK best practice guideline as follows: QD < 5.0, MQ < 40.0, FS > 200.0, and QUAL < 30.0^33^. SNPs of six related species [cat (*Felis catus*, dbSNP build 144), cow (*Bos taurus*, dbSNP build 146), horse (*Equus caballus*, dbSNP build 144), human (*Homo sapiens*, dbSNP build 147), mouse (*Mus musculus*, dbSNP build 146), and pig (*Sus scrofa*, dbSNP build 145)] were collected from dbSNP database^34^. Statistics of sequencing data are provided in Supplementary Table 9.

### SNP annotation and functional analysis of Donggyeong dog-specific genes

SNP annotations of all dog breeds, three wolves, and one coyote were obtained using SnpEff v4.1^21^ with Ensembl gene annotation database for CanFam3.1 dog assembly. Donggyeong dog-specific genes were then identified based on the existence of non-synonymous SNPs present only in Donggyeong dog population (a total of 22 individuals) at orthologous positions compared to other dog breeds and related species. In this step, only non-synonymous SNPs with high or moderate impact predicted by SnpEff, which have potential to affect the loss of function or change protein effectiveness, were used. These gene sets were then analyzed to find overrepresented Gene Ontologies using Panther website^35^ with cutoff Bonferroni-corrected p-value <0.05. Protein sequences corresponding to Donggyeong dog-specific genes were downloaded from the Ensembl Genome Browser^36^. Multiple sequence alignments for protein sequences were performed using MAFFT v7^37^ with default parameters.

### Donggyeong dog subpopulation-specific genes and proteins

Donggyeong dog-specific genes were further classified into two different sets (tail-specific genes and non-tail-specific genes) based on the existence of non-synonymous SNPs in these two different Donggyeong dog subpopulations. For instance, if non-synonymous SNPs of Donggyeong dog-specific genes were only observed from long tail or short tail subpopulation, these genes were classified as tail-specific genes. Non-tail-specific genes were defined similarly. If non-synonymous SNPs of Donggyeong dog-specific genes were observed in both the tail and the non-tail subpopulation, these genes were classified as common genes. For subpopulation-specific genes, the same Gene Ontology enrichment analysis was performed as described above. To perform protein interaction analysis, corresponding protein sets to each of two Donggyeong dog subpopulation-specific gene sets were obtained. Additional proteins interacting with them were added by using evoSNPI pipeline^38,39^. Given a set of input (seed) proteins, evoSNPI calculates the degree of influence, called an interaction score, of all other proteins against the seed proteins over the protein interaction network in the STRING database^40^ by implementing the Random Walk with Restart (RWR) algorithm. Starting from seed nodes in a network, the RWR algorithm explores the given network by either moving to a neighboring node or moving back to the seed nodes with a restarting probability at each step of walk. In this analysis, 0.95 was used as the restarting probability to include proteins close to the seed proteins.

### Protein interaction analysis

Extended Donggyeong dog subpopulation-specific protein sets were used to examine changes in protein interactions in related species (cat, cow, pig, horse, and mouse) based on orthologous protein information acquired from orthoDB^41^. In this analysis, protein-protein interactions with STRING database score of 0.7 or higher were used for credibility. To check the existence of an interaction between two Donggyeong dog proteins in other related species, orthologous proteins of each of these two Donggyeong dog proteins were first identified using orthoDB. There might be multiple orthologous proteins in other species. Therefore, the existence of an interaction in other species was defined only when more than half of all possible pairs of proteins between two orthologous protein groups had interactions. Once the existence or absence of protein interactions in the above related species was determined, ancestral states of protein interactions in common ancestors were predicted by using the maximum parsimony algorithm^42,43^ implemented as an in-house script using the Perl programming language. Based on the existence information of interaction in each descendent species for each pair of proteins, the maximum parsimony algorithm could predict whether the interaction of the same pair of proteins might exist in a target ancestor by minimizing total changes in protein interactions from target ancestor to descendent species. Statistical significance of changes in protein interactions was examined using a null distribution of changes generated from 10,000 random sampling of the same number of protein interactions with the same maximum parsimony algorithm. Visualization and analysis of protein interactions were performed using Cytoscape v3.4.0^44^.

### Data availability

Sequence data generated in this study is available at the NCBI database under BioProject ID of PRJNA417738.

## Electronic supplementary material

Supplementary Figures

Supplementary Table 1

Supplementary Table 2

Supplementary Table 3

Supplementary Table 4

Supplementary Table 5

Supplementary Table 6

Supplementary Table 7

Supplementary Table 8

Supplementary Table 9
