# Supplementary material for: Functional and evolutionary analysis of Korean bob-tailed native dog using whole-genome sequencing data
Source: Sci Rep. 2017 Dec 11;7:17303. doi: 10.1038/s41598-017-17817-w (PMC5725459; doi:10.1038/s41598-017-17817-w)
Supplement: Supplementary file 1 — Supplementary Figures [file 41598_2017_17817_MOESM1_ESM.pdf]

Supplementary Figures

Functional and evolutionary analysis of Korean bob-tailed native dog using whole-genome sequencing data

Daehwan Lee[1,‡], Dajeong Lim[2,‡], Daehong Kwon[1], Juyeon Kim[1], Jongin Lee[1], Mikang Sim[1], Bong-Hwan Choi[2], Seog-Gyu Choi[3], and Jaebum Kim[1,*]

[1]Department of Biomedical Science and Engineering, Konkuk University, Seoul, 05029, South Korea

[2]National Institute of Animal Science, Wanju, 55365, South Korea

[3]Institute of Conservation Gyeongju Donggyeong Dog, Dongguk University, Gyeongju 38170, South Korea

*jbkim@konkuk.ac.kr

‡These authors contributed equally to this work.

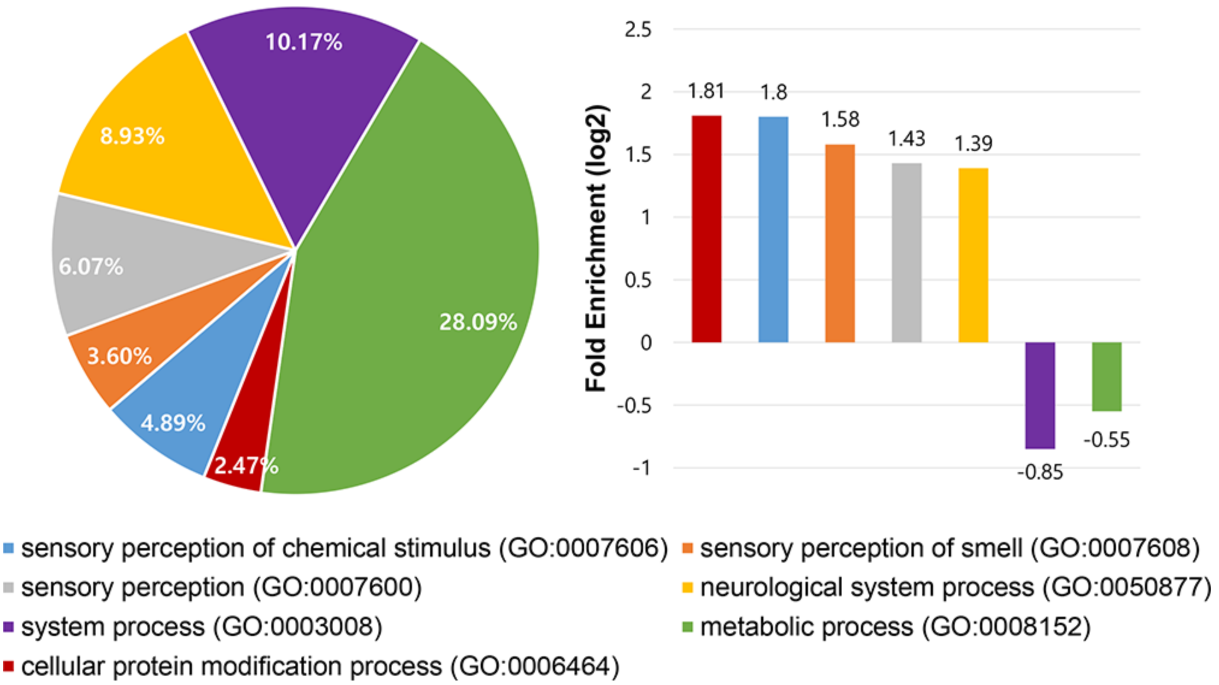

Supplementary Figure 1. Enriched functions in biological process of 1,814 Donggyeong dog-specific genes. The pie chart and bar graph represent the ratio of Donggyeong dog-specific genes in each enriched function and fold enrichment, respectively.

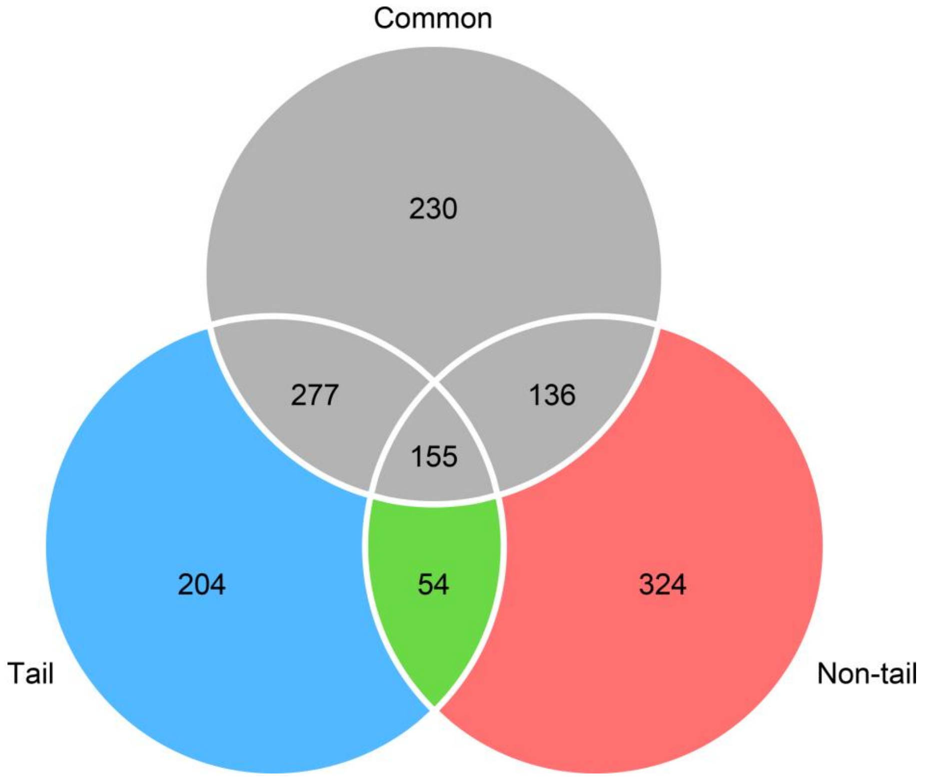

Supplementary Figure 2. Venn diagram of the top 5% proteins in tail-specific, non-tail-specific, and common extended protein sets.
